# ﻿Reinstatement of species rank for *Grimmialimprichtii* (Bryophyta, Grimmiaceae) based on molecular and morphological data

**DOI:** 10.3897/phytokeys.204.82508

**Published:** 2022-08-02

**Authors:** Chao Feng, Jin Kou, Ting-Ting Wu, Guo-Li Zhang

**Affiliations:** 1 College of Grassland, Resources and Environment, Key Laboratory of Grassland Resources, Ministry of Education, China, Key Laboratory of Forage Cultivation, Processing and High Efficient Utilization of Ministry of Agriculture, Inner Mongolia Agricultural University, Hohhot 010011, China Inner Mongolia Agricultural University Hohhot China; 2 School of Life Sciences, Key Laboratory of Molecular Epigenetics of Ministry of Education, Key Laboratory of Vegetation Ecology, Ministry of Education, Northeast Normal University, Changchun 130024, China Northeast Normal University Changchun China

**Keywords:** Asia-Europe disjunction, *Grimmiaobtusifolia*, phylogenetic taxonomy

## Abstract

The genus *Grimmia* Hedw. has been considered taxonomically difficult because of its great morphological variability, and its treatments by different specialists have led to incongruent results. One of the debates in the genus is the species status of *Grimmialimprichtii* Kern, an Asian-European disjunct moss species that has been considered identical to *Grimmiaanodon* Bruch & Schimp. or *Grimmiatergestina* Tomm ex Bruch & Schimp. It has also been regarded as the muticous-leaved male plants of *G.tergestina*. Based on a detailed analysis of the type and many non-type specimens combining the molecular and morphological data, the reinstatement of species rank for *G.limprichtii* is proposed. The diagnostic characteristics of *G.limprichtii* and its distinction from some closely related species, with which it may be confused, are discussed. *Grimmiaobtusifolia* C. Gao & T. Cao is considered a synonym of *G.limprichtii* based on molecular and morphological data.

## ﻿Introduction

The genus *Grimmia* is one of the largest genera of the moss family Grimmiaceae (Feng et al. 2013). Its species are found on all continents, and most of them prefer dry and temperate or cold environments, and all of them are saxicolous with a marked preference for acidic bedrock (Hastings and Greven 2007). Its taxonomy is reputedly difficult because of great morphological variability in most of its species and the difficulty of properly assessing some crucial characteristics (Feng et al. 2014). Therefore, its treatment by different specialists has led to incongruent results (Muñoz 1999; Ignatova and Muñoz 2004). One example is the number of species accepted in the genus, ranging from 51, according to Maier (2010), who synonymized many names of morphologically diverging taxa, to 71, as reported by Muñoz and Pando (2000), to 95, following Hastings and Greven (2007). Some of the controversial species have recently been resolved based on molecular and morphological data (Hugonnot et al. 2018; Kou et al. 2019; Feng et al. 2021).

*Grimmialimprichtii* Kern was described in 1897. However, since it was discovered, this species has been considered identical to *Grimmiaanodon* Bruch & Schimp. (Loeske 1930) and this treatment was accepted by following authors (such as Wijk et al. 1962; Muñoz and Pando 2000). In recent years, it was synonymized with *Grimmiatergestina* Tomm. ex Bruch & Schimp. by emphasizing the cell pattern, structural characteristics of the costa, and characteristics of the perigonial leaves, as well as the occasional presence of both muticous and hair-pointed leaves in the same plant of the latter species (Maier 2002). Soon afterwards, *G.limprichtii* was regarded as the muticous-leaved male plant of *G.tergestina*, as its male plants were associated with sporulating *G.tergestina* in Tibet (Greven 2009).

*Grimmiaobtusifolia* C. Gao & T. Cao was first described in Tibet, China, and later, it was discovered in many other provinces, such as Qinghai, Xinjiang, Sichuan, Tibet of China, and three locations in Mongolia (Tsegmed and Ignatova 2007; Jia and He 2013). In addition, this species may appear in Pakistan (Gruber and Peer 2010). Although *G.obtusifolia* was accepted by some authors (Redfearn et al. 1996; Tan and Jia 1997; Muñoz and Pando 2000; Liu et al. 2011; Jia and He 2013), soon after it was described, *G.obtusifolia* was synonymized by other authors with *G.limprichtii* (Greven and Sotiaux 1995) and *G.tergestina* (Maier 2002, 2010; Greven 2009). Maier (2010) synonymized *G.obtusifolia* with *G.limprichtii* due to similarities in leaf shape, laminal basal cells, and costal architecture, while Greven (2009) believed that *G.obtusifolia* and *G.limprichtii* were muticous-leaved male plants of *G.tergestina*. Plants with muticous leaf apices are not rare in *G.tergestina* and *G.anodon*, and the similar leaf shape, areolation of the leaf base, and costal architecture explain the synonymization with *G.tergestina*, and the nearly unistratose upper laminal cells may explain that with *G.anodon* (Maier 2002, 2010). *Grimmialimprichtii* and *G.obtusifolia* have a similar habit, concave leaves, cucullate and rounded-obtuse leaf apex, architecture of the costa, and areolation of the leaf base. The only difference between the two species is that *G.obtusifolia* has nearly bistratose upper laminal cells, while *G.limprichtii* has unistratose cells with bistratose ridges (Maier 2002; Greven 2014).

Throughout our continuing investigation of xerophilic mosses, which are particularly prevalent in Tibet, many *Grimmia* specimens were collected. Some of them belong to either *G.obtusifolia* or *G.limprichtii*. Detailed observations revealed that these samples bear archegonia, which is contradictory compared to the point of view that *G.obtusifolia* and *G.limprichtii* are muticous-leaved male plants of *G.tergestina*. This discovery prompted us to conduct further morphological and molecular studies to confirm their systematic position.

## ﻿Materials and methods

### ﻿Morphological observations

**Figure 1.**
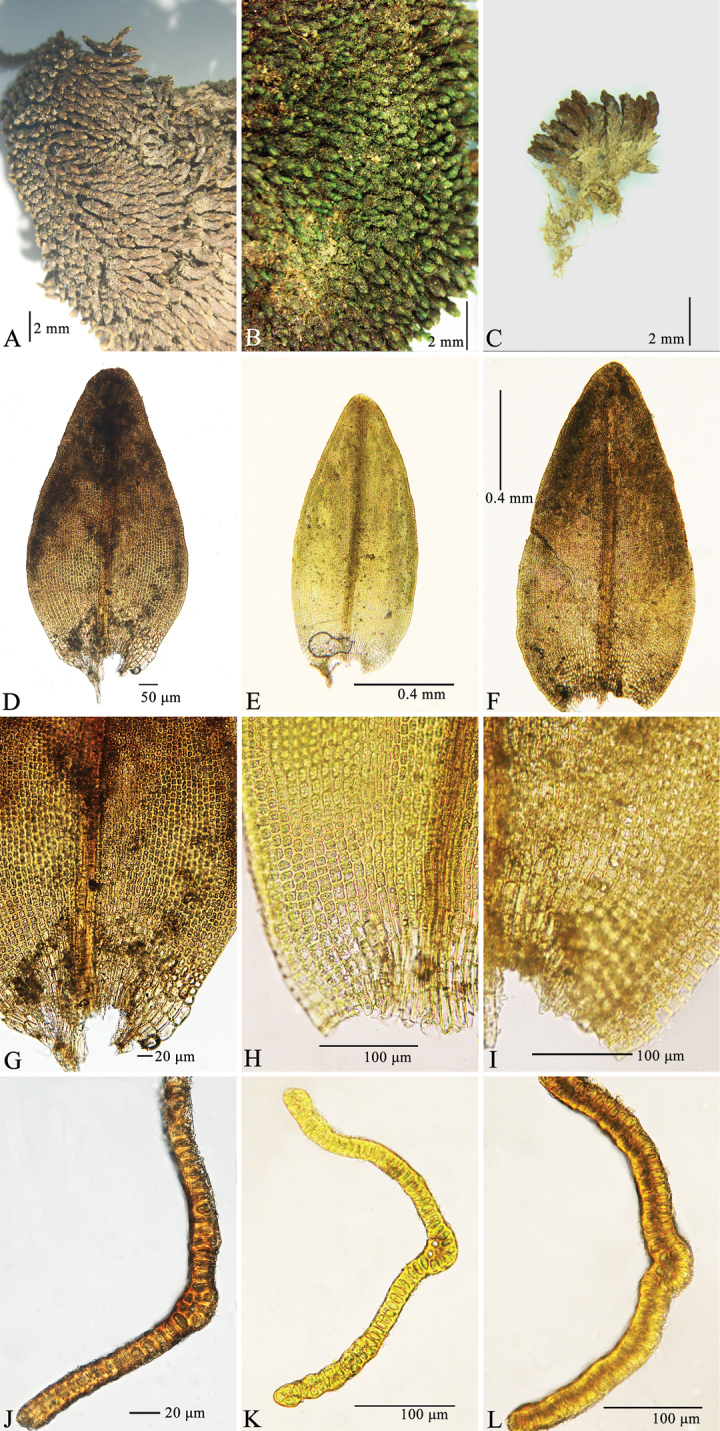
*Grimmialimprichtii***A–C** habit **D–F** leaves **G–I** laminal basal cells **J–L** cross-sections at mid-leaf. [**A, D, G, J** lectotype of *Grimmialimprichtii*, *Kern***B, E, H, K** Tibet, *Zi Wang 20180808022***C, F, I, L** holotype of *Grimmiaobtusifolia*, *Lang 1347*] Photos **A, D, G** and **J** courtesy of the Farlow Herbarium of Harvard University and others by Chao Feng.

Over 2000 specimens of the genus *Grimmia* including types were examined during our revision of Grimmiaceae in China and these specimens were mainly from herbaria investigations (mainly IFP, KUN) and more than 50 field surveys in recent years. All specimens were studied with the typical anatomical and morphological methods applied for the Grimmiaceae (Muñoz 1999; Maier 2010). The collected specimen was deposited at NMAC. Microscopic examinations and measurements were taken with a ZEISS Primo Star light microscope, and microphotographs were obtained with a Canon EOS 70D camera mounted on the microscope. Three plants were dissected from each collection, and for each shoot every possible structure from the gametophyte and sporophyte was examined and a record kept of what was found for each individual species. Specific morphological and anatomical features of taxonomic importance were assessed mainly following Maier (2010) and Muñoz (1999). Leaves were always taken from the upper middle of the stem, and cross-sections were made in the middle part of the stem. Measurements of leaf width were taken at the base, mid- and upper leaf. Cross-sections were made mid-leaf. For comparison the morphological characters of the types of *G.limprichtii*, *G.obtusifolia*, and the sequenced Chinese *G.limprichtii*, the key characters including habit, leaf, laminal basal cells and the cross-sections at mid-leaf of the three specimens were shown in Fig. 1.

### ﻿Taxon sampling, DNA amplification, and sequencing

The only recent collection record from Europe is the material collected in 1993 (Greven and Sotiaux 1995). However, the collection was nearly thirty years ago, which could not be sequenced. To investigate the phylogenetic position of *G.tergestina*, *G.obtusifolia* and *G.limprichtii*, three specimens collected from Tibet were sequenced. Table 1 lists the accessions of the new sequences generated in this study, and Table 2 lists the accessions of the sequences downloaded from GenBank that were used in this study. We employed the nuclear (ITS) marker, which allowed the re-use of earlier results (Streiff 2006; Hernández-Maqueda et al. 2008). DNA extraction, amplification and sequencing of the target regions followed the protocols described in Feng et al. (2021). The PCR products were purified and directly sequenced by the Invitrogen Corporation Shanghai Representative Office. Double-stranded sequencing was performed, and all sequence fragments were edited and assembled using Vector NTI (Suite 11.5) to ensure accuracy.

**Table 1.** New sequences used in this study, including taxa vouchers information and GenBank accession numbers.

Species	Voucher information	ITS	*rps*4	*trnL*-*trnF*
*Grimmiatergestina*_F	China, Tibet, Zi Wang 20180809024	OL514232	OL450501	OL450510
*Grimmialimprichtii*_G	China, Tibet, Zi Wang 20180903002	OL514233	OL450502	OL450511
*Grimmiaobtusifolia*_H	China, Tibet, Zi Wang 20180808022	OL514234	OL450503	OL450512

**Table 2.** Sequences from GenBank used in this study, including taxa and GenBank accession numbers.

Species	ITS	*rps*4	*trn*L–*trn*F
*Coscinodoncribrosus*	–	AJ845205	AJ847855
*Dicranummuehlenbeckii*	–	AF231276	AF231245
*Ditrichumflexicaule*	–	AJ845204	AJ847854
*Drummondiaobtusifolia*	–	AF223038	AF229895
*Dryptodonanomalus*	EU343751	–	–
*Dryptodonaustrofunalis*	EU343752	–	–
*Dryptodondecipiens*	EU343753	–	–
*Dryptodonleibergii*	EU343755	–	–
*Dryptodonpatens*	EU343756	–	–
*Dryptodontorquatus*	EU343757	–	–
*Funariahygrometrica*	–	AJ845203	AJ847853
*Grimmiaalpestris*	–	AJ845237	AJ847887
*Grimmiaanodon*	EU343758	AJ845209	AJ847859
*Grimmiaanomala*	–	AJ845210	AJ847860
*Grimmiaaustrofunalis*	–	AJ845211	AJ847861
*Grimmiabicolor*	EU343759	–	–
*Grimmiacaespiticia*	EU343760	AJ845212	AJ847862
*Grimmiacaespiticia*	EU343761	–	–
*Grimmiacapillata*	EU343762	–	–
*Grimmiacribrosa*	EU343763	–	–
*Grimmiacrinita*	EU343764	AJ845213	AJ847863
*Grimmiadecipiens*	–	AJ845215	AJ847865
*Grimmiadissimulata*	–	AJ845216	AJ847866
*Grimmiadonniana*	EU343765	AJ845217	AJ847867
*Grimmiaelatior*	EU343754	AJ845218	AJ847868
*Grimmiaelongata*	EU343766	AJ845219	AJ847869
*Grimmiafunalis*	EU343767	AJ845220	AJ847870
*Grimmiafunalis*	EU343768	–	–
*Grimmiafunalis*	EU343769	–	–
*Grimmiafunalis*	EU343770	–	–
*Grimmiafuscolutea*	–	AJ845221	AJ847871
*Grimmiahamulosa*	EU343771	–	–
*Grimmiahartmanii*	–	AJ845222	AJ847872
*Grimmiaincrassicapsulis*	EU343772	–	–
*Grimmiaincurva*	EU343773	AJ845223	AJ847873
*Grimmiainvolucrata*	EU343774	–	–
*Grimmiainvolucrata*	EU343775	–	–
*Grimmiakhasiana*	–	AJ845224	AJ847874
*Grimmialaevigata*	EU343776	AJ845225	AJ847875
*Grimmialisae*	–	AJ845226	AJ847876
*Grimmialongirostris*	EU343777	AJ845227	AJ847877
*Grimmiamacroperichaetialis*	EU343778	–	–
*Grimmiameridionalis*	–	AJ845228	AJ847878
*Grimmiamollis*	EU343779	–	–
*Grimmiamontana*	EU343780	AJ845229	AJ847879
*Grimmiamontana*	EU343781	–	–
*Grimmiamuehlenbeckii*	–	AJ845230	AJ847880
*Grimmianevadensis*	EU343782	–	–
*Grimmiaorbicularis*	EU343783	AJ845231	AJ847881
*Grimmiaorbicularis*	EU343784	–	–
*Grimmiaovalis*	EU343785	AJ845232	AJ847882
*Grimmiapilifera*	EU343786	AJ845233	AJ847883
*Grimmiaplagiopodia*	EU343787	AJ845234	AJ847884
*Grimmiapoecilostoma*	EU343788	–	–
*Grimmiapulvinata*	EU343789	AJ845235	AJ847885
*Grimmiapulvinata*	EU343790	–	–
*Grimmiaramondii*	–	AJ845214	AJ847864
*Grimmiareflexidens*	EU343791	–	–
*Grimmiaserrana*	EU343792	–	–
*Grimmiasessitana*	–	AJ845236	AJ847886
*Grimmiatergestina*	EU343793	AJ845238	AJ847888
*Grimmiatorquata*	–	AJ845239	AJ847889
*Grimmiatrichophylla*	–	AJ845240	AJ847890
*Grimmiatrinervis*	EU343794	–	–
*Grimmiaungeri*	EU343795	–	–
*Grimmiaunicolor*	EU343796	AJ845241	AJ847891
*Grimmiawilsonii*	EU343797	–	–
*Hydrogrimmiamollis*	–	AJ845206	AJ847856
*Ptychomitriumgardneri*	–	AF231290	AF231258
*Racomitriumaciculare*	EU343798	AJ845207	AJ847857
*Racomitriumdidymum*	EU343799	–	–
*Racomitriumelongatum*	EU343800	–	–
*Racomitriumheterostichum*	EU343801	–	–
*Schistidiumapocarpum*	–	AJ845208	AJ847858
*Schistidiumcrassipilum*	EU343802	–	–
*Schistidium* sp. ‘*lingulatum*’	EU343750	–	–
*Scouleriaaquatica*	–	AF306984	AF231179

### ﻿Phylogenetic analyses

The sequences were aligned using MAFFT 7.222 (Kazutaka and Daron 2013) and then edited in BioEdit 7.0.1 (Hall 1999). The concatenation of each individual *rps*4 and *trn*L-*trn*F fragments was performed using our custom Perl script. Phylogenetic analyses were performed using Bayesian inference (BI) and maximum likelihood (ML). MrBayes 3.2.6 (Ronquist and Huelsenbeck 2003) was used for BI analyses under the GTR substitute model. Two Markov Chain Monte Carlo (MCMC) searches were run for 1 million generations each, with a sampling frequency of 1000. The first 25% of the trees were discarded as burn-in. A posterior probability (PP) of 0.95–1.00 was considered strong support. The convergence between runs in all cases dropped below 0.01. ML analyses were executed in IQ-TREE 1.6.3 (Nguyen et al. 2014) under the TPM2u+F+G4 (for cpDNA) and TIM+F+I+G4 (for ITS) substitute models, respectively, selected by the ModelFinder program (Kalyaanamoorthy et al. 2017) based on the Bayesian information criterion (BIC), and 1000 fast bootstrapping replicates were used. Nodes with bootstrap (BS) values of 70–89% were treated as moderate and 90–100% as well supported. The final tree obtained was visualized and edited in FigTree v.1.4.0 (Rambaut 2014).

## ﻿Results

### ﻿Molecular data

**Figure 2.**
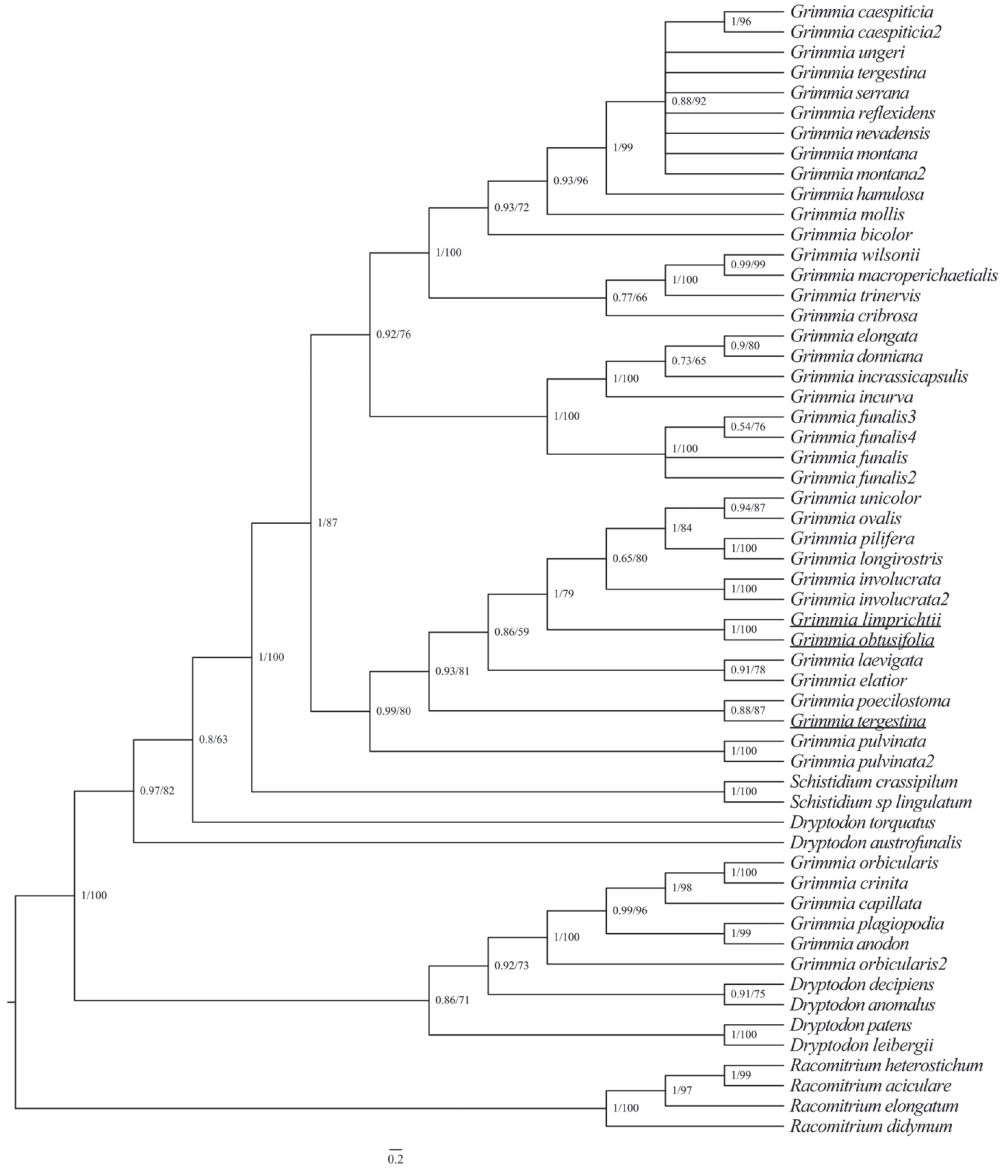
Phylogenetic relationships (50% majority consensus tree) from the Bayesian inference on the ITS dataset. Numbers above branches indicate posterior probability from the BI analysis, followed by bootstrap values for the ML analysis. The species investigated in this study were marked in underscore.

**Figure 3.**
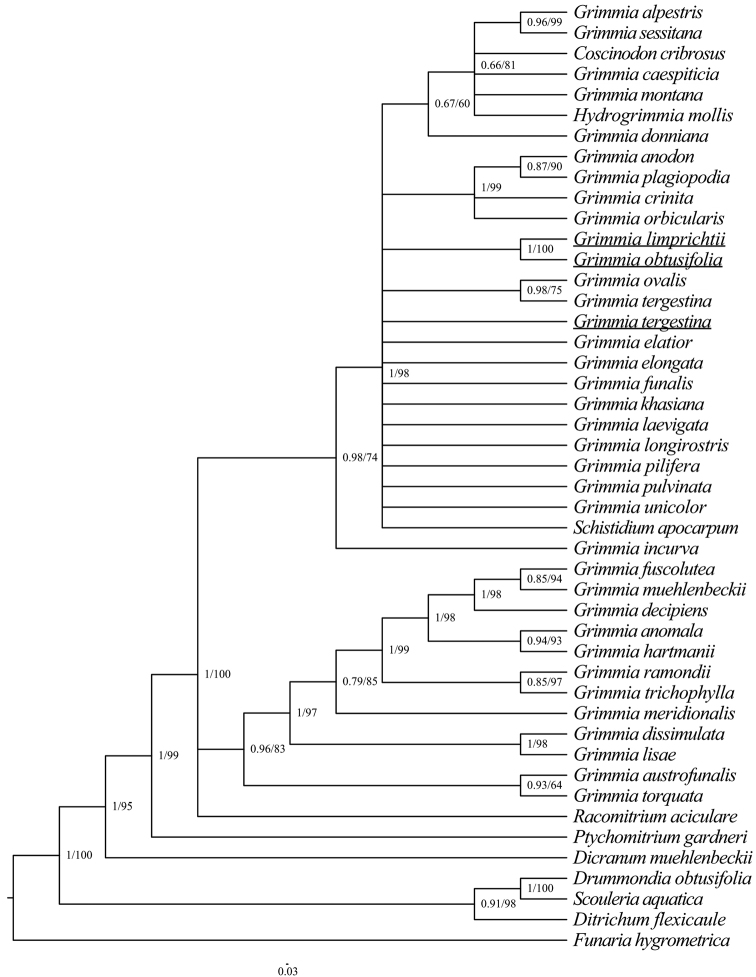
Phylogenetic relationships (50% majority consensus tree) from the Bayesian inference of the concatenated *rps*4 and *trn*M-*trn*V datasets. Numbers above branches indicate posterior probability from the BI analysis, followed by bootstrap values for the ML analysis. The species investigated in this study were marked in underscore.

The chloroplast (cp) and ITS alignments comprised 1149 and 1509 nucleotide sites, respectively. The BI and ML phylogenetic trees had a consistent topology, although there were different levels of support depending on the method. Hence, only the topology with branch lengths from the BI tree is presented, with added support from the ML method on the respective trees (Figs 2, 3). The inference from ITS (Fig. 2) and the chloroplast regions (Fig. 3) agree in most aspects. The topology of both ITS data and chloroplast data resolved *G.limprichtii* and *G.obtusifolia* as sister taxa in a strongly supported clade (BS = 100, PP = 1). *Grimmialimprichtii* and *G.obtusifolia* are not closely related to *G.tergestina*.

### ﻿Taxonomic treatment

#### ﻿ Grimmia limprichtii

Taxon classificationPlantaeGrimmialesGrimmiaceae

Kern , Revue Bryologique 24: 56. 1897.

97CB26D0-6DB5-5B28-B2C8-EDACDD537980

**Figure 4.**
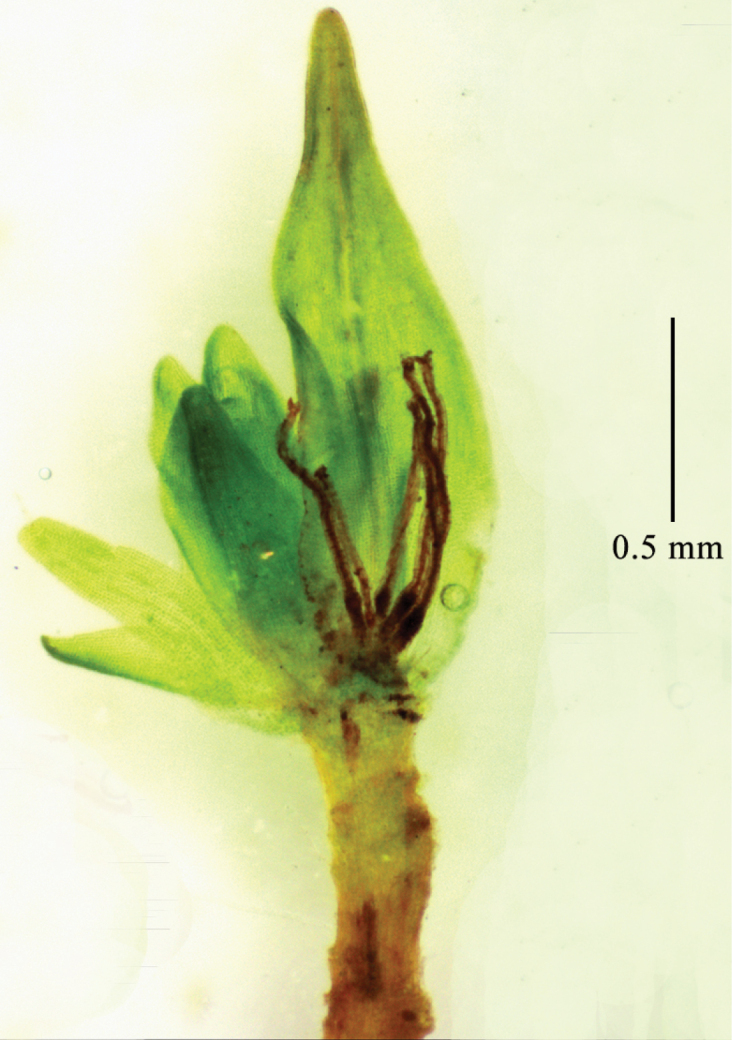
*Grimmialimprichtii* archegonia. Photos: Chao Feng (*Zi Wang 20180808022*).

Figs 1 , 4 Chinese name: 林氏紫萼藓

Grimmia obtusifolia C. Gao & T. Cao, Acta Botanica Yunnanica 3: 394. f. 4: 10–16. 1981. Type: Tibet, Shuanghu Xian, Lang 1347 (holotype: IFP!; paratypes: IFP!, MO).

##### Type.

Dolomiten, Palagrouppe: Felsgallerien am limone, bei 2100m. 29.7.96 Kern (lectotype: FH!; isolectotypes: Goet!, JE, PC).

For full description and illustration, see Cao and Vitt (1986), Greven and Sotiaux (1995), and Feng (2014).

## ﻿Discussion

*Grimmialimprichtii* is a remarkable species characterized by small and slender plants, muticous, concave to somewhat keeled and oblong-ovate leaves, somewhat cucullate and rounded-obtuse leaf apex, plane leaf margins, and a costa ending below the apex. In addition, its sexual condition is dioicous. Although the androecia of *G.limprichtii* were discovered in Europe and Asia (Greven and Sotiaux 1995), its archegonia were usually found in our collections from Inner Mongolia (Feng 2014) and Tibet (Fig. 4), but androecia were not found. Our findings showed that the presumption that *G.limprichtii* is the muticous-leaved male plant of *G.tergestina* (Greven 2009) is unreliable. The generation of a single generative organ in a specific area may explain why the sporophytes are not generated. The characteristic bistratose, partially bistratose or unistratose with bistratose ridges in the upper part of laminal cells is an intraspecific variation influenced by ecological factors, based on our molecular and morphological results.

Morphologically, *G.limprichtii* is most similar to *G.tergestina*, a widely distributed species (Muñoz 1999; Ignatova and Muñoz 2004). Both species share similar leaf shapes, plane leaf margins, and indistinct costa. Additionally, some specimens of the latter species are found in leaves both with and without hair-points (Maier 2002). However, *G.limprichtii* can be readily distinguished from *G.tergestina* by its small and slender plants, costa ending below the apex, and costal guide cells in laminal parts that are distinct from laminal cells. While *G.tergestina* has rather stiff plants, costa percurrent and guide cells of the laminal part of the costa are hardly distinct or even indistinct from lamina cells, due to their similarity.

*Grimmiacrassiuscula* H.C.Greven & C.Feng, a species that was recently described from the Helan mountains, China (Greven and Feng 2014), resembles *G.limprichtii* in the oblong-ovate and muticous leaves, cucullate leaf apex, plane leaf margins, and costa ending below the apex. Nevertheless, *G.crassiuscula* differs from *G.limprichtii* in having plants in loose and succulent mats, absence of a central strand of the stem, and costa without stereids.

*Grimmialimprichtii* was previously synonymized with *Grimmiaanodon* Bruch & Schimp., a widely distributed species (Muñoz 1999; Hastings and Greven 2007). Although hair-point presence and length and the number of cell layers in leaf cross sections are variable in the latter species (Muñoz 1999), *G.anodon* can be separated readily from *G.limprichtii* by its keeled and broadly oblong-lanceolate leaves, elongate-rectangular laminal basal cells, and autoicous sexuality. *G.limprichtii*, by contrast, has concave and oblong-ovate leaves, quadrate to rectangular laminal basal cells, and dioicous sexuality.

## Supplementary Material

XML Treatment for Grimmia limprichtii
